# Enhancing Animal Disease Resistance, Production Efficiency, and Welfare through Precise Genome Editing

**DOI:** 10.3390/ijms23137331

**Published:** 2022-06-30

**Authors:** Zhiguo Liu, Tianwen Wu, Guangming Xiang, Hui Wang, Bingyuan Wang, Zheng Feng, Yulian Mu, Kui Li

**Affiliations:** 1 State Key Laboratory of Animal Nutrition, Key Laboratory of Animal Genetics Breeding and Reproduction of Ministry of Agriculture and Rural Affairs of China, Institute of Animal Sciences, Chinese Academy of Agricultural Sciences, Beijing 100193, China; liuzhiguo@caas.cn (Z.L.); wutianwen@caas.cn (T.W.); guangming202012@163.com (G.X.); wang2728037554@163.com (H.W.); wangbingyuan@caas.cn (B.W.); 2 Guangdong Provincial Key Laboratory of Animal Molecular Design and Precise Breeding, Key Laboratory of Animal Molecular Design and Precise Breeding of Guangdong Higher Education Institutes, School of Life Science and Engineering, Foshan University, Foshan 528231, China; greatfz@126.com; 3 Agricultural Genome Institute at Shenzhen, Chinese Academy of Agricultural Sciences, Shenzhen 518124, China

**Keywords:** livestock animals, genome editing, precision breeding, disease resistance, regulatory system

## Abstract

The major goal of animal breeding is the genetic enhancement of economic traits. The CRISPR/Cas system, which includes nuclease-mediated and base editor mediated genome editing tools, provides an unprecedented approach to modify the mammalian genome. Thus, farm animal genetic engineering and genetic manipulation have been fundamentally revolutionized. Agricultural animals with traits of interest can be obtained in just one generation (and without long time selection). Here, we reviewed the advancements of the CRISPR (Clustered regularly interspaced short palindromic repeats)/Cas (CRISPR associated proteins) genome editing tools and their applications in animal breeding, especially in improving disease resistance, production performance, and animal welfare. Additionally, we covered the regulations on genome-edited animals (GEAs) and ways to accelerate their use. Recommendations for how to produce GEAs were also discussed. Despite the current challenges, we believe that genome editing breeding and GEAs will be available in the near future.

## 1. Introduction

Genetic improvement of economic traits, such as disease resistance, meat production, and meat quality, is the main focus of animal breeding. Through selective breeding and crossbreeding, some economic traits, such as growth and reproduction, have been greatly enhanced over the past several decades. However, these conventional breeding methods have been costly and painstakingly slow, and some polygenetic traits, such as disease resistance, have not been dramatically improved. The development of large animal genetic manipulation technology, particularly CRISPR (Clustered regularly interspaced short palindromic repeats)/Cas (CRISPR associated proteins) system mediated genome editing, has provided efficient ways to affect traits of interest to produce agricultural animals in just one generation.

The CRISPR/Cas system has led to a revolution in the field of genetic manipulation and tremendously expanded its range, providing great tools for animal biotechnology research and livestock breeding. Rapid advancements have been made not only in gene editing, base editing [1], and prime editing [2] but also in transcriptional regulation and post-transcriptional engineering using CRISPR/Cas system-based tools. In recent years, CRISPR/Cas tools have revolutionized the field of large animal breeding. They have shown great promise not only for reducing selection time and production costs, but also for improving characteristics difficult to achieve or not amenable by traditional breeding methods. Valuable traits such as disease resistance, meat production, meat quality, and traits that can improve animal welfare can now be efficiently achieved by CRISPR/Cas tools. In this review, we systematically presented the innovations in CRISPR/Cas genome editing tools and their application in agricultural animal breeding. We also discussed some of the challenges encountered with genome-edited animals (GEAs) and its future applications.

## 2. CRISPR/Cas System Mediated Genetic Manipulation

**Figure 1:**
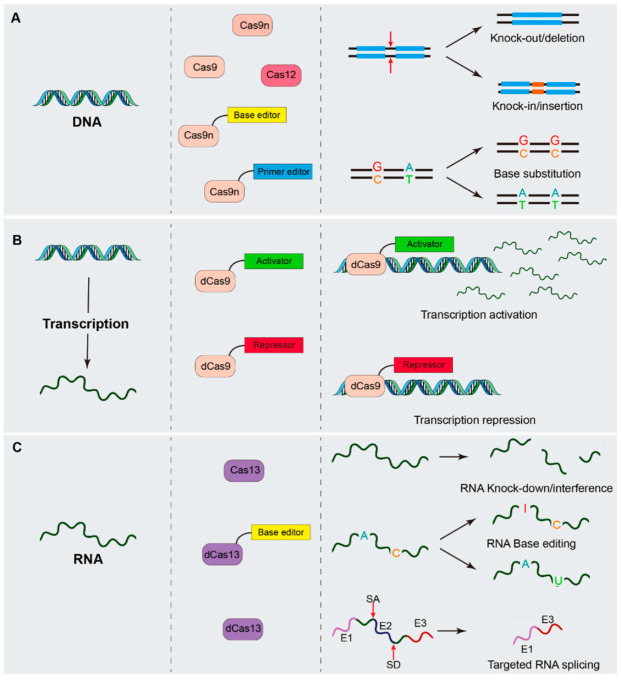
CRISPR/Cas systems mediated genetic manipulation. CRISPR-Cas systems allow multiple levels of genetic manipulation. (**A**) At the DNA level, Cas9, Cas9n and Cas12a are used for inducing dsDNA breaks for knock-out/deletion or knock-in/insertion. Cas9n can also be fused to base editors or primer editor to modify nucleotides in dsDNA for base substitution or base rewriting without introducing a dsDNA break. (**B**) During transcription, dCas9 can be fused to transcriptional activators, repressors, or epigenetic modifiers to activate or repress the transcription of single or multiple genes. (**C**) At the level of RNA, Cas13 can be used for targeted RNA manipulation. Cas13 can knock down specific RNA molecules by catalyzing RNA cleavage. Cas13 fused to base editors can be used to modify nucleotides in RNA molecules to achieve RNA base substitution or base rewriting. A single or multiple crRNA that bind to splice site motifs such as SA and SD combined with dCas13 protein can block specific exon from recognition by the splicing machinery, resulting in targeted RNA splicing. E1, exon 1; E2, exon 2; E3, exon 3; SA, splice acceptor site; SD, splice donor site.

Precision genomic DNA modification can now be achieved with much greater simplicity and efficiency using the CRISPR/Cas system (Figure 1A). RNA-guided Cas9, Cas9 nickase (Cas9n), and Cas12 can cleave the genome in a specific position resulting in double-stranded breaks (DSBs), which trigger the cell to repair the DNA damage by either non-homologous end-joining (NHEJ) or homology-directed repair (HDR). NHEJ can introduce small insertions or deletions, HDR can introduce a targeted mutation with a donor sequence. A base editor can perform precise point-mutations without DSBs; it consists of a Cas9n and nucleobase deaminase enzyme that catalyzes targeted deamination reactions. Several different base editors have been developed so far [2]: cytosine base editors (CBEs) catalyze C > T transitions, adenosine base editors (ABEs) facilitate transitions of A > G, guanosine base editors can attain C > G transversions [3], and dual adenine and cytosine base editors [4,5] can achieve C > T and A > G conversions at the same time.

Another alternative gene-editing technology named ‘prime editing’ has further expanded the range of precision genomic DNA modification [6]. Prime editors not only can catalyze all twelve possible transition and transversion mutations but can also mediate small insertion or deletion mutations. It consists of a Cas9n fused with a reverse transcriptase (RT) domain and a pegRNA (prime editing guide RNA). The pegRNA not only directs primer editor binding to the target DNA sequence and then facilitates nicking of the target strand, but also works as a template for the RT domain to synthesize a new DNA strand. This new strand, which contains the modification site, is then inserted into the target site following DNA repair.

Beyond genomic DNA modification, CRISPR/Cas system can be used to activate or repress RNA transcription (Figure 1B). Catalytically deficient Cas9 (dead Cas9, dCas9) can be used as a modular platform, coupling with transcriptional regulators or domains, such as VP64, KRAB [7,8], or DNMT3A [9], can attain specific, rapid, and multiplexed transcriptional activation, repression, or epigenetic modification in a range of cell types [10,11].

At the post-transcriptional level, gene expression regulation can also be manipulated by CRISPR/Cas system (Figure 1C). Class 2 type VI Cas proteins Cas13 family have programmable RNase activity [12,13,14], which can cleavage the RNA molecular by the binding of a guide RNA. Therefore, Cas13 proteins have been employed in RNA interference [12], targeted RNA splicing [12,15], and RNA base editing [16,17] in mammalian cells. It has been proved that Cas13 proteins can efficiently and specifically mediate mRNAs and long non-coding RNAs (lncRNAs) knock-down in mammalian cells [12], embryos [18], and mouse models [19,20]. Furthermore, catalytically inactivated Cas13 (dead Cas13, dCas13) protein fused with splicing regulatory domains can be directed by a crRNA to activate or perturb specific exons [12,15]. Dead Cas13 fuses with nucleobase deaminase enzymes instead of Cas9n, and can be used to edit a single RNA base in cells [16,17], providing a new method for correcting point mutations at the post-transcriptional level.

## 3. Disease Resistance Breeding

One of the most important applications of genome editing on farm animals is to improve the resistance or tolerance to pathogens. Farm animal infectious diseases not only cause huge economic losses to the animal husbandry industry but also threaten human health. It has always been an insurmountable industrial problem that bothers animal breeders and veterinary experts. However, disease resistance is a complex and polygenic trait; using traditional genetic selection for disease resistance breeding is much more costly, time-consuming, and inefficient. Moreover, the broad application of vaccines and antibiotics weakened the urgency of disease resistance selection programming to a certain extent. But transgenic and gene targeting have been successfully used in breeding antiviral animals such as cattle free of prion protein [21] and pigs that express anti-foot-and-mouth disease virus (FMDV) shRNA [22]. Recently, genome editing technology, especially CRISPR/Cas mediated gene knock-out/knock-in and precise modification, highly improved the efficiency of disease resistance animal breeding.

Leukotoxin secreted by *Mannheimia (Pasteurella) haemolytica* binds to the uncleaved signal peptide of CD18 protein and causes cytolysis of ruminant leukocytes [23], resulting in acute inflammation and lung tissue damage, inflicting a huge economic loss to the world-wide cattle industry. The first-generation gene editing tool zinc finger nuclease (ZFNs) have been applied to introduce a single amino acid mutation in the bovine CD18 protein [24]. Leukocytes from the *CD18*-gene-edited cattle expressed CD18 protein without the signal peptide, thus these leukocytes were resistant to leukotoxin-induced cytolysis. Another example is the *CD163* gene. Porcine reproductive and respiratory syndrome (PRRS) is a worldwide infectious disease that costs million dollars a year to the swine industry. Due to the genetic diversity of PRRS virus (PRRSV), vaccines have not been able to control the disease. In 2010, Van Breedam et al. identified that *SIGLEC1* (also known as *CD169*) and *CD163* were necessary surface receptors for PRRSV entrance and uncoat in porcine cells [25,26]. Based on this finding, *SIGLEC1* knock-out pigs [27] and *CD163* knock-out pigs [28,29] were generated by CRISPR/Cas technology. A follow-up study on *CD163* biallelic knock-out pigs showed that they process full resistance to PRRSV [30], no matter when they were infected directly or exposed to infected pen mates. Further studies show that *CD163* knock-out pigs are also completely resistant to the infection of highly pathogenic PRRSV, which is more virulent than classical type 2 PRRSV [31].

However, *CD163* gene has a variety of biological functions, knock-out may have a negative physiological impact on the economic traits of pigs. Therefore, a further precision modification was performed by either replacing the domain with orthologous CD163 protein domain [32,33] or by deleting SRCR domain 5 [34,35,36,37]. These studies providing a basis for further investigation of the essential region or even several amino acids associated with PRRSV infection. Once the essential amino acids for PRRSV infection were identified, by changing only one or several amino acids, PRRSV-resistant pig lines that retaining the biological functions of CD163 protein can be obtained. This might be achieved by the application of base editing technologies and tools in the near future.

Similar to *CD163*, porcine aminopeptidase N (*pAPN*, also known as *ANPEP*, *CD13*) gene was identified as a candidate receptor of transmissible gastroenteritis virus (TGEV) and porcine epidemic diarrhea virus (PEDV). Inhibition or direct knock-out of *pAPN* in cells can moderate TGEV infection [38,39]. Pigs with null *pAPN* are resistant to TGEV, but retained susceptibility to infection with PEDV [40,41]. Xu et al. (2020) successfully generated *CD163* and *pAPN* gene knock-out pigs using CRISPR/Cas9 and somatic cell nuclear transfer (SCNT) [42]. These double-gene-knock-out (DKO) pigs not only exhibit complete resistance to both PRRSV and TGEV but also exhibit decreased susceptibility to porcine deltacoronavirus (PDCoV) infection. Furthermore, there are no differences in the production performance, reproductive performance, or pork nutrient content between DKO pigs and wild-type control pigs. This study shows that multiple sites editing in a pig genome is feasible by CRISPR/Cas9 and cellular screening, and also enlightened that breeding animals with multiple desirable traits, like disease-resistant, can be achieved by cellular surface receptors editing. In another research, Tu et al. (2019) obtained CMP-N glycolylneuraminic acid hydroxylase (*CMAH*) gene knock-out pigs by microinjection of two single-guide RNA and Cas9 mRNA. Although *CMAH* knock-out piglets exhibited delayed PEDV onset and diminished disease severity, they are not immune to PEDV [43]. Therefore, there is still a lot of work to be carried out in order to find the PEDV receptor.

Besides gene knock-out, site-specific expressing of resistance-associated genes could also be a strategy to breed disease resistance livestock. Bovine tuberculosis, which is caused by *Mycobacterium bovis*, is a serious threat to the agricultural economy and human health. Currently, no effective programs exist to control bovine tuberculosis in many less-developed areas of the world. The mouse *SP110* (also known as *Ipr1*) gene can limit the growth of *Mycobacterium* in macrophages and inducing apoptosis in infected cells [44]. Research proved that integrating the mouse *SP110* gene into the bovine genome by TALEN can control the growth of *Mycobacterium* and limit the transmission of tuberculosis in pen mates [45]. The natural resistance-associated macrophage protein-1 gene (*NRAMP1*, also known as *SLC11A1*) of bovine is associated with innate resistance to intracellular pathogens such as *Mycobacterium*, *Leishmania*, *Salmonella*, and *Brucella*. Adding a copy of bovine *NRAMP1* gene to the specific locus of bovine genome by single Cas9 nickase can provide cattle with increased resistance to tuberculosis [46]. Histone deacetylase 6 (*HDAC6*) has anti-viral activities during the viral life cycle, overexpressing *HDAC6* enhances the resistance to PRRSV infection both in vitro and in vivo [47].

**Figure 2:**
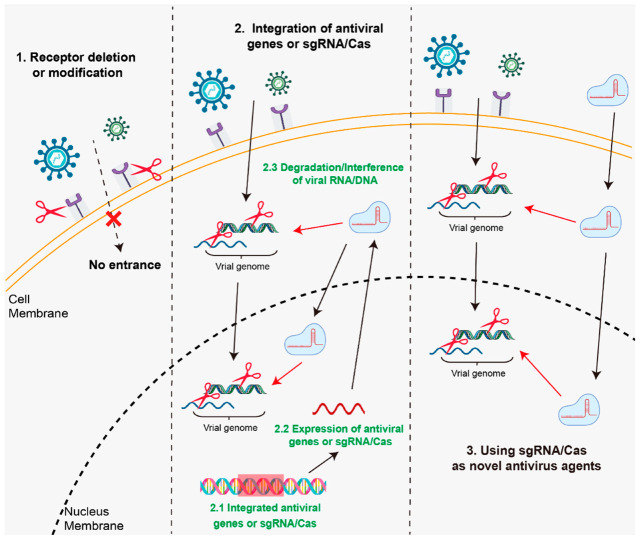
Various antiviral strategies mediated by CRISPR/Cas systems. (**1**) Receptor deletion or modification: To enter the host cell, certain viruses use cell membrane receptors. CRISPR/Cas technologies can eliminate or modify these receptor genes, preventing virus-receptor binding and providing full resistance to animals. (**2**) Integration of antiviral genes or sgRNA pools and Cas9 or Cas13 proteins into animal genomes: Using the CRISPR/Cas technique, single or multiple antiviral genes or sgRNA pools and Cas9 or Cas13 genes can be integrated into the safe harbors of animal genome, the constant expression or spatio-temporal specific expression of these genes will interfere with or degrade viral RNA or DNA. (**3**) Using sgRNA/Cas as novel antivirus agents: sgRNAs polls targeting multiple viral sequences together with Cas9 or Cas13d proteins can be delivered to animal cells via viral delivery systems (Lentivirus or adeno-associated virus system) or non-viral delivery systems (Nanoparticles) to interfere with or degrade viral RNA or DNA.

As CRISPR/Cas system is the adaptive and heritable immune system of bacteria and archaea. It is reasonable that CRISPR/Cas system could also be used as a designed immune system to eliminate or inhibit the replication of animal viruses. This strategy was already tested in animal cells. Tang et al. (2017) designed 75 single guide RNAs (sgRNA) targeting both essential and nonessential genes across the genome of pseudorabies virus (PRV) [48], which is a swine herpesvirus that causes significant economic losses in the worldwide swine industry, in vitro experiments found that most of the sgRNAs significantly inhibited PRV replication. More importantly, they also demonstrated that targeting PRV with sgRNA pools that contain multiple sgRNAs can completely abolish the production of infectious viruses in cells. African swine fever (ASF) is another economically important infectious disease of swine with high mortality rates, which threatens pig production across the globe. It is caused by the African swine fever virus (ASFV), which is a double-stranded DNA virus, and no essential cell surface receptors have been identified. Hubner et al. (2018) observed complete abrogation of ASFV yields by targeting the viral phosphoprotein p30. However, they also found ASFV mutants with one or two nucleotides can escape CRISPR/Cas9 inhibition [49]. These data proved the possibility that Cas9 and multi-targeting sgRNA could be developed as an efficient antiviral strategy. With multiple sgRNAs that targeting multiple viral sequences, Cas9 or Cas13d can be directed to recognize and degrade viral DNA or RNA, providing broad-spectrum antiviral capabilities for animals (Figure 2). Cas9 or Cas13d and sgRNAs could be integrated into the genome of transgenic animals to breed antiviral animals, or delivered to animal cells as novel antivirus agents.

However, there seem to be many hurdles to overcome before its realization. First, it is necessary to evaluate the therapeutic potentials in vivo, and a detailed analysis of the host immune response to the Cas9 and Cas13 proteins and multiple sgRNAs needs to be conducted for the prediction of potential side effects associated with this antiviral therapy. Second, the potential off-target activity of this CRISPR/Cas9-based antiviral therapy needs to be evaluated, especially when multiple sgRNAs were employed to degrade the viral genome. Various measures including high-infidelity Cas9 variants should be taken to minimize the potential off-target activity before in vivo application. Third, the cost of this CRISPR/Cas9-based antiviral therapy needs to be fairly inexpensive to applicate on farms, at least it should be cost-effective than biosafety measures that are already widely used in animal farms to control ASFV, PRV, PRRSV, etc. Despite these challenges, the full healing potential of CRISPR/Cas system based antiviral therapy should be able to motivate its development.

## 4. Improving Production Performance

Increasing animal lean meat rates is the mainstream breeding goal of food animals for a very long time. *Myostatin* (*MSTN*, also known as *GDF8*) is a negative regulator of skeletal muscle mass [50]. Natural mutations of *MSTN* have been reported in cattle [51,52], sheep [53], dogs [54], pigs [55,56,57], and human [58]. These animals show a double-muscled phenotype of dramatically increased muscle mass. In adult tissues, *MSTN* is expressed almost exclusively in skeletal muscle, but detectable levels of *MSTN* RNA are also present in adipose tissue [59]. *MSTN* knock-out confers a remarkable increase in lean meat yield [60,61,62] in many mammals and increased levels of polyunsaturated fatty acids in pigs [62,63]. Although *MSTN* knock-out significantly increased lean meat production of pigs, severe hindlimb weakness was observed among *MSTN^−/−^* newborns in Western commercial pig breeds [55,64]. This congenital hindlimb weakness defect is a prohibitive bottleneck to the safe and ethical application of *MSTN*-editing in pigs. Fan et al. (2021) performed a long-term and multidomain evaluation for multiple *MSTN*-edited pig breeds. They demonstrated a practical alternative edit-site-based solution to overcome the hindlimb weakness and illustrated that *MSTN*-editing can sustainably increase the yields of breed-specific lean meat and the levels of desirable lipids without deleteriously affecting feed-conversion rates or litter size.

Another strategy to overcome the hindlimb weakness is to mimic the naturally existing *MSTN* mutations, such as the Piedmontese c.G938A mutation and the Belgian Blue mutation (821del11). The Piedmontese c.G938A mutation at the *MSTN* results in the substitution of a highly conserved cysteine to tyrosine (p.C313Y) in the mature region of the protein. Wang et al. (2016) introduce a missense point mutation, which mimicking the orthologous p.C313Y mutation, and generated one cloned piglet harboring the p.C313Y mutation via SCNT [65]. Zou et al. (2019) introduced an 11-bp deletion, which is orthologous to the natural Belgian Blue *MSTN* mutation, at the exon 3 of pig *MSTN* gene and obtained two cloned Duroc piglets [66]. In addition, site-specific insertion of *MSTN* inhibitor was also performed to enhance the growth performance [67]. These works expand the range of modifying *MSTN* gene, holding great promise for animal breeding and disease modeling.

Another potential candidate gene for improving meat production in pigs is the insulin-like growth factor 2 (*IGF2*) gene. *IGF2* regulates cellular proliferation, differentiation, and apoptosis in both fetal and post-natal growth. The transcription and expression of *IGF2* are downregulated by the zinc finger BED domain-containing protein 6 (*ZBED6*), mutations in the *IGF2* intron 3-3072, which is a *ZBED6* binding site, can upregulate the expression of *IGF2* and improve muscle development [68]. Modifying this ZBED6 binding site by CRISPR/Cas9 genome editing tools will greatly enhance the muscle development in indigenous Chinese pig breeds such as the Chinese Bama pig and the Liang Guang Small Spotted pig [69,70]. In sheep, the suppressor cytokine signaling 2 (*SOCS2*) gene plays a vital role in the control of bone mass and body weight. A point mutation g.C1901T in *SOCS2* is highly associated with increased body weight and size in sheep [71]. Zhou et al. (2019) obtained gene-edited lambs with a C to T point mutation in *SOCS2* gene by micro-injection of programmable deaminases BE3 into sheep zygotes and without inducing unintended off-target mutations at the genome-wide scale [72].

The *fat-1* gene is a fatty acid desaturase gene that originates in *Caenorhabditis elegans*. In the last two decades, a large number of *fat-1* transgenic animals have been developed to convert n-6 polyunsaturated fatty acids (n-6PUFAs) to n-3 polyunsaturated fatty acids (n-3PUFAs) and increase meat quality. However, the random integration transgene strategy frequently results in fluctuating transgene expression and the insertion of selected marker genes. Li et al. (2018) used the CRISPR/Cas9 technology to introduce a single copy of the *fat-1* gene into the porcine *Rosa26* locus, resulting in site-specific *fat-1* knock-in pigs with a considerable rise in n-3PUFAs levels [73]. Zhang et al. (2018) used CRISPR/Cas9 to insert the *fat-1* gene into the goat *MSTN* locus, resulting in simultaneous deletion of endogenous genes and site-specific *fat-1* gene knock-in [74]. You et al. (2021) created double-gene knock-in pigs by inserting single copies of the *fat-1* and *IGF-1* genes into the porcine *Rosa26* locus at the same time [75]. These pigs have a great potential for boosting pork’s nutritional value.

Uncoupling protein 1 (*UCP1*) is a key element of nonshivering thermogenesis and is important for preventing cold and regulating body adiposity. However, domestic pigs lack a functional *UCP1* gene, making them susceptible to cold and prone to fat deposition. Zheng et al. (2017) used a CRISPR/Cas9-mediated approach to efficiently insert mouse *UCP1* cDNA into the porcine endogenous *UCP1* locus [76] and obtained *UCP1*-KI pigs, which had less fat deposition, higher carcass lean percentage, and, most importantly, improved ability to maintain body temperature in cold environments. These pigs provide a potentially valuable resource for farm animal production.

## 5. Improving Animal Welfare

Genome editing in livestock will not only improve farm animals’ resistance or tolerance to pathogens, increase production performance, but also will prevent unnecessary animal suffering, which may encourage public support of GEAs for food chain production.

In modern livestock, daily management of horned cattle poses a high risk of injury for each other as well as for the farmers. Physical dehorning of cattle is used to protect animals and farmers from accidental injury but is associated with stress and pain for the calves. Naturally occurring structural variants causing hornlessness are known for most beef cattle. The polled Celtic variant from the genome of an Angus cow was integrated into dairy cattle using genome editing tools and somatic cell cloning [77]. The presence of some substances in male pork such as androstenedione and methylindole will make the ‘boar taint’ and affect the taste of pork. To improve pork quality and to facilitate production management, boars are usually castrated after birth. In 2016, the Australian Society of Animal Production (ASAP) conference presented a new method of using CRISPR/Cas9 technology to knock-out the *KISSR* gene (responsible for testicular development in pigs) to block testicular development and achieve the effect of depopulation [78]. Taken together, these research works show that genome editing is precise, sustainable, and directly applicable to improved animal well-being.

In summary, with the rapid development of genome editing tools, GEAs with desirable features can now be efficiently obtained (Table 1). Before these GEAs are commercialized, their breeding potential and/or safety should be assessed. 

## 6. Regulations on GEAs

The growth of green and sustainable agriculture to feed the world’s growing population will be substantially aided by GEAs, but how to control GEAs and genome-edited animal products remains an unsolved dilemma. As a result of the technological breakthroughs, regulations created for the control of transgenic animals appear to no longer be adequate for GEAs.

On 14 December 2020, the U.S. Food and Drug Administration (FDA) approved GalSafe^TM^ pigs for medical and/or food use, it is the first-of-its-kind intentional genomic alteration (IGA) in livestock [87]. On 7 March 2022, FDA announced it has made a low-risk determination for the marketing of products, including food, from two “PRLR-SLICK” genome-edited beef cattle and their offspring after determining that the intentional genomic alteration does not raise any safety concerns [88]. The IGA results in the equivalent genotype (genetic make-up) and short-hair coat trait seen in some conventionally bred cattle, known as a “slick” coat. This is the FDA’s first low-risk determination for enforcement discretion for an IGA in an animal for food use. On 24 January 2022, Ministry of Agriculture and Rural Affairs of China officially issued the “Guidelines for Safety Evaluation of Gene Edited Plants for Agricultural Use (for Trial Implementation)”, marking a significant simplification of China’s regulatory policy on the safety evaluation of gene edited plants, which is expected to further accelerate the industrialization of biological breeding in China [89]. In countries such as Argentina, Australia, and Brazil, regulation is not required if genome-edited animals do not contain any foreign DNA [90,91].

Realizing the enormous potential of GEAs for long-term solutions to serious environmental and food security challenges will be the first step toward allowing their broad usage in most countries in the world. Governments, developers, manufacturers, and consumers must all work together and communicate effectively. For governments, a case-by-case GEA review and regulatory method will result in a system that is more efficient, objective, complete, and operational [92,93]. For developers, using DNA-free editing techniques to create GEAs, such as gRNA/Cas9 ribonucleoproteins (RNP) [94] and base editor RNP, should help gain public trust.

## 7. Prospects and Challenges

Genome editing technology offers ground-breaking tools and methodologies for manipulating the genomes of large animals, opening up new possibilities for livestock breeding and animal husbandry. It is expected that other applications will be developed, and that genome-edited livestock-derived meat will be available for consumption in the near future. However, when producing animals for agriculture, the off-target effect remains a key concern. Off-target mutations may result in knock-out events or silent mutations in protein-coding genes, or interference with transcriptional regulation. Mutations in protein-coding regions may cause aberrant form of proteins, which may induce food allergenicity. Changes in translational regulation may have an impact on animal health, reproduction, and growth performance. Therefore, low-risk genome editing methods, such as DNA-free genome editing, have gained much attention recently. DNA-free genome editing strategies could profoundly reduce the risk of off-target mutations [94]. Somatic cell nuclear transfer (SCNT) could also be used to eliminate off-target mutations before GEAs breeding. By the SCNT approach, it is possible to verify the genotype and off-target mutations of donor cells before live animal production takes place. It can also avoid the occurrence of genetic mosaicism and reducing the overall cost of genome edited livestock production. These aspects are especially critical for application in large domestic animals that have particularly long generation intervals.

Another trend is to use base editor tools to create point mutation in large animal instead of Cas9 tools. Base editing tools have several advantages over Cas9 tools. The first one is that base editors do not cause DSBs. DSBs caused by Cas9 has been shown to result in excessive DNA damage and cell death, using base editor tools instead of Cas9 in animal breeding can avoid these unpredictable risks, especially when editing multiple genes simultaneously. Additionally, Base editors have higher efficiency in making point mutations. For example, the efficiency of making point mutation at porcine *MSTN* gene by Cas9 and single-stranded oligonucleotides mediated HDR was 10.3% [64], while 46.3% target point mutation were achieved at porcine *MSTN* locus by using an optimized cytosine base editor (hA3A-BE3-NG) [95]. Furthermore, by only one transfection, a three genes base-editing rate of 71.4% can be achieved at the porcine *GGTA1*, *B4galNT2*, and *CMAH* loci [96]. This research indicates that base editors are more efficient in achieving point mutations, especially for multiple gene editing.

In summary, the rapid progress of gene editing technologies will greatly accelerate their application in domestic animal husbandry. GEAs with valuable traits will contribute to the goal of developing green and sustainable agriculture for the world.

## Figures and Tables

**Table 1:** List of genome-edited livestock animals.

Species	Gene	Modification *	Method *	Applications	References
Pig	*RELA*	KO	ZFN	Disease resistance	[79]
	*CD169*	KO	HR	Disease resistance	[27]
	*CD163*	KO	CRISPR/Cas9	Disease resistance	[29,30,31]
	*CD163* (SRCR 5 domain)	*hCD163L1* SRCR domain 8 replacement	CRISPR/Cas9	Disease resistance	[32,33]
	*CD163* (SRCR 5 domain)	Domain deletion	CRISPR/Cas9	Disease resistance	[34,35,36]
	*HDAC6*	TG	SCNT	Disease resistance	[47]
	*CD163*	Partial domain deletion	CRISPR/Cas9	Disease resistance	[37]
	*pAPN*	KO	CRISPR/Cas9	Disease resistance	[40]
	*CD163, pAPN*	Double KO	CRISPR/Cas9	Disease resistance	[42]
	shRNA	TG	Injection	Disease resistance	[22]
	*CMAH*	KO	CRISPR/Cas9	Disease resistance	[43]
	*IGF2*	Regulatory element mutation	CRISPR/Cas9	Meat production	[69,70]
	*IRX3*	KO	CRISPR/Cas9	Fat content	[80]
	*FBXO40*	KO	CRISPR/Cas9	Meat production	[81]
	*PBD-2*	KI	CRISPR/Cas9	Disease resistance	[82]
	*MSTN*	Point mutation	CRISPR/Cas9	Meat production	[65]
	*MSTN*	Partial deletion	CRISPR/Cpf1	Meat production	[66]
	*FST*	KI	CRISPR/Cas9	Meat production	[67]
	*MSTN*	KO	CRISPR/Cas9	Meat production	[62]
	*RSAD2*	KI	CRISPR/Cas9	Disease resistance	[83]
	*fat-1*	KI	CRISPR/Cas9	Meat quality	[73]
	*fat-1, IGF1*	Double KI	CRISPR/Cas9	Meat quality and meat production	[75]
	*UCP1*	KI	CRISPR/Cas9	Fat content and animal welfare	[76]
	*KISSR*	KO	CRISPR/Cas9	Animal welfare	[78]
Cattle	*SP110*	KI	TALENs	Disease resistance	[45]
	*NRAMP1/SLC11A1*	KI	CRISPR/Cas9	Disease resistance	[46]
	*CD18*	Point mutation	ZFN	Disease resistance	[24]
	*IARS*	KI	CRISPR/Cas9	Animal welfare	[84]
	*POLLED allele*	KI	TALENs	Animal welfare	[77]
Sheep	*BMPR1B*	Point mutation	CRISPR/Cas9	Reproductive traits	[85]
	*MSTN*	KO	CRISPR/Cas9	Meat production	[60]
	*SOCS2*	Point mutation	Base Editor	Growth rate	[72]
Goat	*GDF9*	KI	CRISPR/Cas9	Reproductive traits	[86]
	*fat-1, MSTN*	KI and KO	CRISPR/Cas9	Meat quality and meat production	[74]
	*MSTN*	KO	CRISPR/Cas9	Meat production	[61]

* KO, knock out. KI, knock in. TG, transgene. HR, homologous recombination.

## Data Availability

Not applicable.
